# Biosynthesis of a VLP-type nanocarrier specific to cancer cells using the BEVS expression system for targeted drug delivery

**DOI:** 10.1186/s43141-023-00479-9

**Published:** 2023-02-16

**Authors:** Mohammad Sadegh Hashemzadeh, Nariman Gharari

**Affiliations:** 1 Nanobiotechnology Research Center, Baqiyatallah University of Medical Sciences, Tehran, Iran; 2 Department of Molecular Biotechnology and Health Sciences, University of Turin, Turin, Italy

**Keywords:** CPV-VLP, BEVS system, VP2, *Sf9* cell, Baculovirus

## Abstract

**Objective:**

Canine parvovirus (CPV) is a small virus without an envelope that consists of three viral proteins including VP1, VP2, and VP3. Exclusively, the VP2 can form a typically CPV-sized virus-like particle (CPV-VLP) that can be used as a biological nanocarrier for diagnostic and therapeutic purposes since these VLPs can target cancer cells specially through the transferrin surface receptors (TFRs). Consequently, we aimed to produce these nanocarriers to be used for specific targeting of cancer cells.

**Methods:**

*Sf9* insect cells were transfected with constructed recombinant bacmid shuttle vector encoding an enhanced green fluorescent protein (EGFP) and CPV-VP2 by the cationic lipids of Cellfectin II. Subsequently, two recombinant baculoviruses expressing EGFP and VP2 were produced and expression of VP2 was increased under the optimal condition. In consequence, the CPV-VLP nanoparticles composed of recombinant VP2 subunits were extracted. The purity of VLPs was then evaluated by SDS-PAGE, and the structural integrity and quality of the final product were evaluated by TEM and HA methods. Eventually, the size distribution of the produced biological nanoparticles and their uniformity were determined by the DLS method.

**Results:**

The expression of EGFP protein was confirmed by fluorescent microscopy, and the expression of VP2 protein was evaluated by SDS-PAGE and western blotting. Infected *Sf9* insect cells also showed cytopathic effects (CPEs), and the maximum expression of VP2 occurred at MOI of 10 (pfu/cell) at the harvest time of 72 h post-infection (hpi). After performing various stages of purification, buffer exchange, and concentration, the quality and structural integrity of the VLP product were confirmed. The results of the DLS technique showed the presence of uniform particles (PdI below 0.5) with an approximate size of 25 nm.

**Conclusion:**

The results indicate BEVS as an appropriate and efficient system for generating CPV-VLPs, and the used method based on two-stage ultracentrifugation was appropriate for purifying these nanoparticles. Produced nanoparticles can be used as the biologic nano-carriers in future studies.

## Background

Canine parvovirus (CPV) is a small virus approximately 26 nm in diameter, without an envelope and a natural pathogen of dogs consisting of three viral proteins (VPs) including VP1, VP2, and VP3. The VP2 protein is the major component of the viral capsid and binding ligands for CPV to enter the host cell through interaction with the transferrin surface receptors (TFRs). The importance of CPV virus-like particles (CPV-VLP) in binding to human cancer cells motivated researchers to generate these proteins. Most likely, the expression of VP2 alone as the main part of the capsid, surface epitopes, and ligands results in the assembly of typically-sized VLP. Therefore, VLPs can be used as biological nanocarriers for diagnostic and therapeutic purposes [1, 2]. After binding to TFRs, natural CPV enters the cytoplasm through VP2 protein with the mechanism of clathrin-mediated endocytosis. Inside the endosome, the decrease in environmental acidity leads to changes in the viral capsid. As a result, the viral capsid protein VP1 could release the virus from the endosome into the cytoplasm by its phospholipase A2 (PLA2) activity. Through the microtubule and dynein in the cytoplasm, the viral capsid is transferred to the nucleus. Virus localization to the nucleus, where the virus replicates, requires nuclear localization sequence which is present in the VP1 [3]. In our study, we have used the AcMNPV baculovirus shuttle vector that is commonly used in biotechnology industries which is 80–200kb and consists of polyhedrin and p10 promoters inserted in a circular vector.

The natural receptors for CPV are called transferrin receptors. Transferrin is an iron-carrying protein which strongly required during cell growth. Parvovirus particles can use these receptors to enter the cell; however, since humans consider ineffective to these viruses, they are unable to replicate in human cells. If the transferrin receptor expression is normal, the viral particles cannot enter the cell, while the cancer cells require a considerable amount of iron to grow, and the transferrin receptor is expressed in large amounts. Thereby, the viral particles can enter the cells. Once the virus enters the cell, due to the lack of signals from the nucleus, the next steps for the virus to replicate are not supported and it remains inside the cytoplasm [3].

Transferrin (TF) is an iron-binding protein that is strongly required during cell growth [4]. Since cancer cells require an impressive amount of iron to grow, the transferrin receptor is highly expressed and presented in various types of cancer cells. Paradoxically, the expression of transferrin receptors in normal cells is extremely low. As a result, high expression of transferrin receptors is considered one of the markers in order to identify cancer cells. The CPV can bind to these receptors and enters the cells; consequently, it is expected that canine parvoviral particles (CPV-VLPs) will be suitable nanoparticles for identifying tumor cells, especially their metastases, and targeting them for drug or gene therapy [4, 5].

The VLPs have a wide range of applications as a biological nanoparticle. These nanoparticles are one of the best tools for the generation of the next-generation viral vaccines as well as drug carriers in cancer therapy, especially the VLP, which naturally contain high affinity for cancer cells, and it is due to their simple generation compared to reverse genetic systems [3, 6–8]. They are also suitable alternatives for high-risk agents, killed and attenuated live vaccines that purify the toxic agents in the generation processes. Additionally, their structure is unknown compared with the basic structure; however, for VLP, it is necessary to form a basic and expected structure since necessary subunits are used to form basic ones. Another application of these nanoparticles contains employment as nano-particles to present the main epitopes to the immune system. These VLPs can also act as nano-carriers for drug or gene delivery or delivery of the desired molecules to the target cell specifically [3, 9–11].

## Methods

As mentioned previously [12, 13], first, we generate a mini-Tn7 transposon containing the eukaryotic expression cassette of the *VP2* gene in the pFastBac1 donor plasmid vector by two steps of cloning. Then, the recombinant pVP2FastBac1 plasmid was transferred into *Escherichia coli* DH10Bac competent cells and the recombination of the *VP2* gene was accomplished into baculovirus-derived bacmid shuttle vector using the Bac-to-Bac system. The *EGFP* gene was coupled with the VP2 gene in all steps parallelly, as a control [13, 14]. The constructed recombinant bacmids including *VP2* and *EGFP* genes were analyzed by PCR panel, using designed primers and PUC/M13 universal primers and then transfected into *Sf9* insect cells using cationic lipids to generate two new recombinant baculoviruses expressing EGFP and CPV-VP2 (Ethic NO.IR.BMSU.REC.1400.037) [15].

### Cell culture

*Sf9* insect cells originated from clone No. 9 of ovarian cells cultured by the larvae of *Espodoptera frogiperda*. Mycoplasma negative cells attach well to the surface of the glass or flask and form monolayer cells, doubling in number every 18 to 24 h (peak growth rate).

### Recombinant expression of EGFP and CPV-VP2 and optimization of expression conditions in insect cells

Following the preparation of high-grade virus seeds on a large scale, in order to optimally express the recombinant protein, it is necessary that *Sf9* insect cells with a confluency of 80–85% in the presence of 2% serum become infected with the recombinant virus with high MOI (1–10 pfu/cell). In the baculoviral expression vector system (BEVS), the maximum expression for secretory proteins is between 30 and 72 h post-infection (hpi) (preferably using the *high five* cell, which is a derivative of the *Trichoplusia* ni cell line) and for non-secretory proteins 48 to 96 hpi (preferably using *Sf9* and *Sf21* cells).

Determining the optimal conditions for protein production depends on various factors such as insect cell type, the type of target protein, and the environment used, so both virus MOI and the time of protein harvest on different days should be evaluated.

To perform the experiment, 15 flasks of T25 containing single-layer cell culture were prepared with an initial rate of 5×10^5^ cells per milliliter of culture medium supplemented with 2% bovine serum albumin, and when the cells reached a confluency of 80–85%, slightly higher than the logarithmic half-growth, they were inoculated with two different recombinant viruses expressing VP2 and EGFP separately. In order to perform the experiment, the flasks were divided into three groups of five. Two groups were infected with MOIs 1 and 10 and one group was selected as cell control and all were incubated at 27 ± 0.5° C. One flask from each group was harvested at 24, 48, 72, 96, and 120 hpi and compared with control cells for morphology and density. The cells of each harvested flask, after scraping, were centrifuged at low speed and washed with cold PBS at least 1–2 times (until the cells were not suffered or dead) to completely remove the serum. The possibility of washing for adherent cells before padding is not possible for all flasks, because after infection, the cell attachment becomes weaker over time and many cells are even detached, and if the supernatant is removed and the cells are washed, a large portion of the cells are virtually removed, which reduces the expression of total rate.

The final precipitate was suspended in 100 μl of PBS buffer and to prevent degradation of the released proteins, PMSF anti-protease with a final concentration of 1 mM was added to the suspension and the samples were stored at −20 ° C until next use.

After homogenization, all collected samples were first concentrated by the Bradford method to examine the protein product (total protein as well as the target recombinant protein content) to determine the changes in total protein content in each sample), then a specific volume (e.g., 10 μl) of each sample was electrophoresed on a polyacrylamide gel to evaluate and compare changes in total protein as well as target recombinant protein content in different samples.

It should be noted that the volume parameter was fixed, and changes in protein concentration in the samples were examined. The best MOI and harvest time with the highest product quality were selected and used to produce the desired protein in a large scale.

### Increasing the expression of recombinant VP2 under the optimal conditions

After optimizing the harvest time of inoculated *Sf9* insect cells and selecting the best MOI, *Sf9* cells were cultured in at least 30 flasks of T75 and inoculated with recombinant viruses under optimal conditions to produce recombinant CPV-VP2 protein and EGFP. The infected cells were then removed from the flask and collected in 50 ml falcons and centrifuged at 3000 rpm for 4 min at 4 °C. After washing twice with cold PBS in order to completely remove the serum and culture medium, the cell precipitate was stored in the −20 °C until the extraction and purification of CPV-VLP, or it was kept at −70 °C by adding the appropriate amount of extraction buffer and transferring to cryotube. It should be considered that whether the serum is not completely removed, in the case of SDS-PAGE with the range of 55–65 kDa, protein bands that interfere with the examined protein appears, so the necessity of removing by proper washing should take into account carefully.

### The extraction and purification of the formed VLPs

There are various methods for extracting and purifying VLP produced in insect cells, which are mainly based on the use of sucrose or cesium chloride gradient and ultracentrifugation because VLP is a particle and this is one of the salient features of VLPs. Therefore, it has special floating and sedimentation density. The floating density of CPV natural capsid is about 1.33 g/cm^3^, its sedimentation rate is between S110 and S122, and its molecular weight is about 5×10^6^ to 2.6×10^6^ g/mol, which is similar to CPV-VLP characteristics. In ultracentrifugation with a sucrose or cesium chloride gradient, different proteins are separated based on their specific buoyancy density. Due to the gradient solution concentration in the centrifuge tube, the density is lower on top of the tube and higher at the bottom. The cell suspension is transferred into a tube, and during centrifugation, each cell component extract is fractionated at a specific level in the tube. In continuous gradient concentration, the concentration changes along the centrifuge tube, and in the presence of large components, an infinite concentration band may form. In the discontinuous gradient concentration, there will be different concentrations and a limited number of levels along the centrifuge tube. In addition, a component may still not be perceptible due to its extremely low density, even at very high cycles and very low-density environments, and may not have the so-called particle properties.

### Investigation of the structural integrity of the purified VLPs with transmission electron microscopy (TEM)

The morphology and structural integrity and quality of the purified VLP were examined using transmission electron microscopy (TEM) and negative staining. For this purpose, 10 μl of purified VLP sample was placed on a special carbon grade and after 1 min, a large amount of solution was taken with the help of filter paper. Afterwards, 10 μl of 1% (up to 2.5) uranyl acetate stain solution was transferred to the grid and after 1.5 min, a large amount of solution was taken with filter paper and kept to dry at room temperature. After drying, the grades were evaluated by the Hitachi Transmission Electron Microscope (Model HU12-A) with a magnification of 120,000×.

### Evaluation of the quality and structural integrity of the purified VLPs by hemagglutination assay (HA)

CPV has a strong ability to agglutinate the red blood cells of chicken. But VP2 protein monomers alone lack this property. Since the VLP of CPV whether formed, has a structure similar to the normal capsid, it is expected to have similar hemagglutination properties. Therefore, hemagglutination assay (HA) with chick red blood cells (RBCs) was used to evaluate the structural integrity and quality of purified VLP.

### Preparation of RBC 1%

Some blood was collected from 2- to 3-week-old chicken wings and transferred into a microtube containing 300 μl of PBS and 1 μl of anticoagulant heparin (5000 U/ml). The sample was centrifuged at 1500 rpm and 4 °C for 5 min, the supernatant was gently drained with a Pasteur pipette, and the remaining red blood cells were washed several times with cold PBS until the supernatant was completely clear. Finally, 50 μl of washed RBCs was added to 5 ml of cold PBS and 1% RBC suspension was created.

A 100μl suspension containing 500ng of purified VLP was used, and two-fold dilutions were made in a row of 96 wells plate with a final volume of 50 μl in the PBS buffer, so that in all wells (except the first well), 50 μl of PBS buffer was transferred and 50 μl of the 100 μl protein suspension of the first well was removed and added to the second well. This dilution was performed again by removing 50 μl from the second well and adding to the other wells, respectively. Afterwards, 50 μl of RBC 1% is prepared from chicken blood added to each well and placed at 4 °C for 3 h. In a row, as a negative control, 50 μl of RBC 1% was added with 50 μl of PBS. Moreover, RBC should not be transferred from the start point. Since repeated pipetting during the dilution process leads to the lysis of red blood cells, meanwhile, the dilution of RBC is also disturbed.

During incubation, the VLP forms a network with red blood cells. Therefore, after the end of the above time, in the wells containing appropriate amounts of VLP, there is no sediment formation observed. Additionally, in negative control wells and also wells containing VLP less than the required amount to form a network, red blood cell deposition is observed as a red dot at the bottom wells.

### Investigation of the size distribution of formed nanoparticles by dynamic light scattering (DLS) technique

In order to evaluate the size distribution of formed nanoparticles, the dynamic light scattering (DLS) technique was used (Malvern Zeta-sizer). The numbers obtained were calculated based on the loaded protein amount and density in the sample, by the relevant software. The results of CPV-VLP particle size analysis showed that 68.8% of the formed nanoparticles had a size of 25.12 nm and 31.2% have a size of 184.4.

## Results

### Evaluation of the cells containing the baculovirus encoding EGFP protein with an inverted fluorescent microscope

**Fig. 1:**
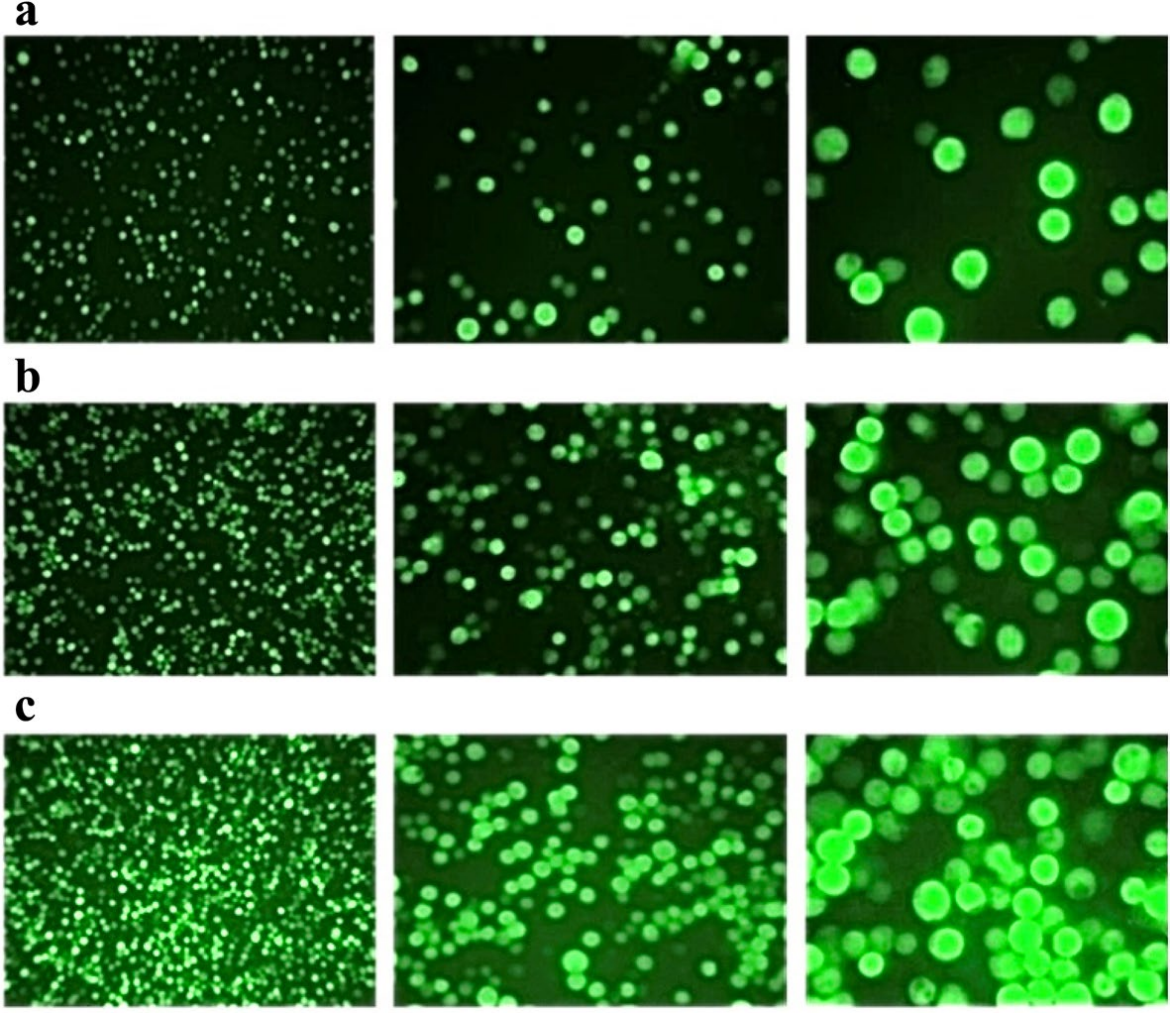
The *Sf9* insect cells infected by the recombinant baculoviruses expressing EGFP under the inverted fluorescent microscope by three magnifications of 10×, 20×, and 40× from left to right. **a** The cells transfected by recombinant bacmid shuttle vector (P0), **b**, **c** The cells infected by P1and P2 baculoviral seeds, respectively (*P* passage)

As demonstrated in the previous studies [12–15], first we generated two baculoviruses expressing VP2 and EGFP proteins. The expression of recombinant EGFP protein in the cells infected with recombinant baculoviruses encoding the *EGFP* gene was observed by an inverted fluorescent microscope. With this technique, small amounts of the EGFP protein were clearly visible in non-optimal conditions. The expression of this protein indicated the accuracy of the baculovirus expression system as a control and the relevant processes (Fig. 1).

### Estimation of viral titer by sequential dilutions method

**Fig. 2:**
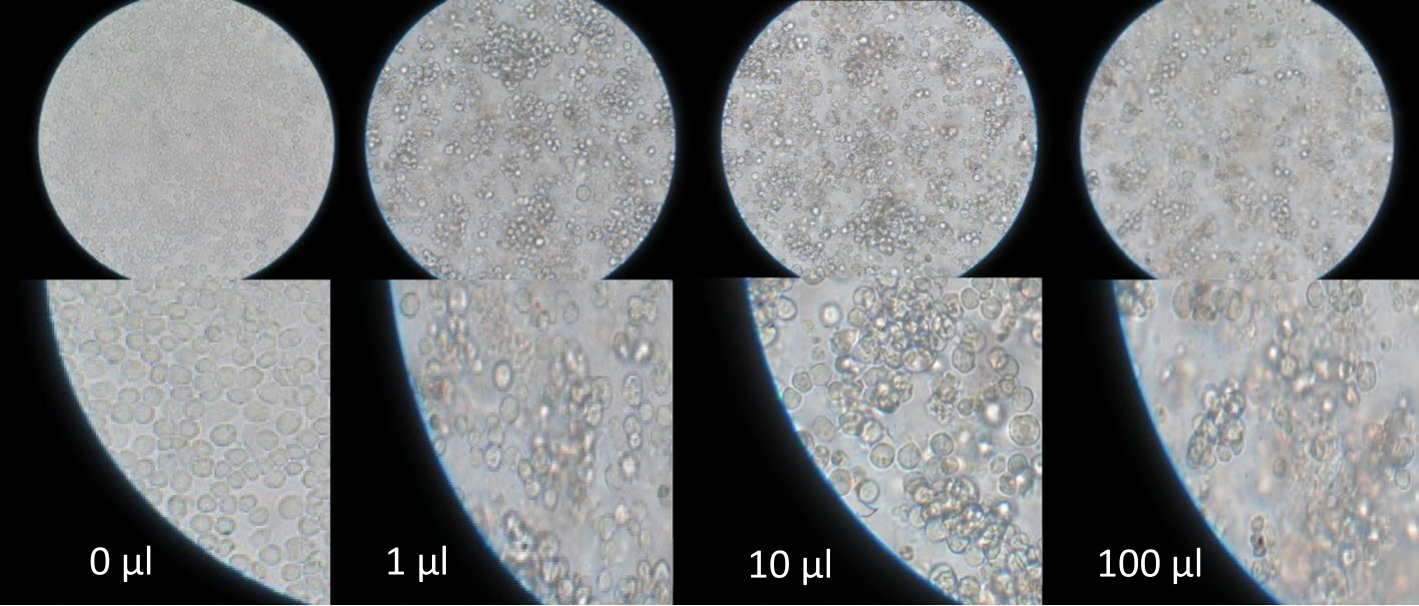
Estimation of viral titer by sequential dilutions method. The *Sf9* insect cells inoculated with 1, 10, and 100 μl of P3 baculoviral seed showed almost the same CPEs and thus had the titer of 2×10^8^ pfu/ml

Due to the difficulty to work with the baculovirus system and also the plaques resulting from recombinant baculovirus infection are difficult to observe, the sequential dilutions method was used to estimate the amount of produced recombinant viruses. The results showed that the cells inoculated and infected with 1, 10, and 100 μl of P3 baculoviral stock had almost the same CPEs (Fig. 2) and thus had the titer amount of 2×108 pfu/ml. While P2 baculoviral stock had almost the same results in inoculation with only 10 and 100 μl .P1 baculoviral stock showed not the same results. As a result, the titer of the first generation (P1), the second generation (P2), and the third generation (P3) of recombinant baculoviruses were determined to be the coefficient of 10^6^, 10^7^, and 10^8^ pfu/ml, respectively. In order to collect the virus from culture to prepare a viral stock, the suitable MOI is 0.1–1 pfu/cell for inoculation and the number of passages should not exceed P3. Moreover, after preparing high-amount viral seeds, in order to express the recombinant protein, it is necessary to infect *Sf9* cells with a recombinant virus by high MOI (1–10 pfu/cell). Since in case of virus inoculation with low MOI (0.1–1 pfu/cell), a 10-fold increase in titer of the next generation compared to inoculated viral titer, is reasonable and expected. Consequently, the first, second, and third generations of the baculoviruses obtained had a sufficient quality for storage as the viral stocks.

### Expression of recombinant VP2 in insect cells at two different MOIs

The Bradford assay was used to measure the total protein content in each flask. The amount of total protein in the cell sediment obtained from the culture in a T25 flask inoculated with recombinant baculovirus with MOI of 1 (pfu/cell) at 24, 48, 72, 96, and 120 h was equal to 463.3 μg, 481.5 μg, 524.6 μg, 582.2 μg, and 541.2 μg, respectively, and with MOI of 10 (pfu/cell) at 24, 48, 72, 96, and 120 h was equal to 488.1 μg, 443.4 μg, 408.5 μg, 365.2 μg, and 273.8 μg, respectively. However, the amount of the total protein in cells from the control flasks (not inoculated with the virus) at 24, 48, 72, 96, and 120 h was equal to 710 μg, 1018 μg, 1488 μg, 1422 μg, and 1403 μg, respectively. As can be observed, the total protein content in the control flasks increased with the growth of cells and the increase in the number of them and then decreased with the onset of cell death and the decrease in the number of cells.

In cells infected with MOIs of 1 and 10 (pfu/cell), the total protein content showed a decrease compared with the first-day control flask, indicating that growth stopped in cells infected with recombinant baculovirus and protein synthesis and initiation of recombinant protein expression of virus (thus reducing the expression of other proteins), while during the first 24 h, the control cells continue to proliferate. Over time, the expression of recombinant VP2 and consequently the total protein content in infected cells with MOI of 1 (pfu/cell) increased and from the fourth day onwards, by increasing cell death, the total and recombinant protein content decreased. Despite the fact that cell death occurs in infected cells in the initial days, there is also cell growth in non-infected cells and background expression in SDS-PAGE is almost constant until the fourth day; furthermore, increasing the protein content until the fourth day is due to increased expression of recombinant protein.

**Fig. 3:**
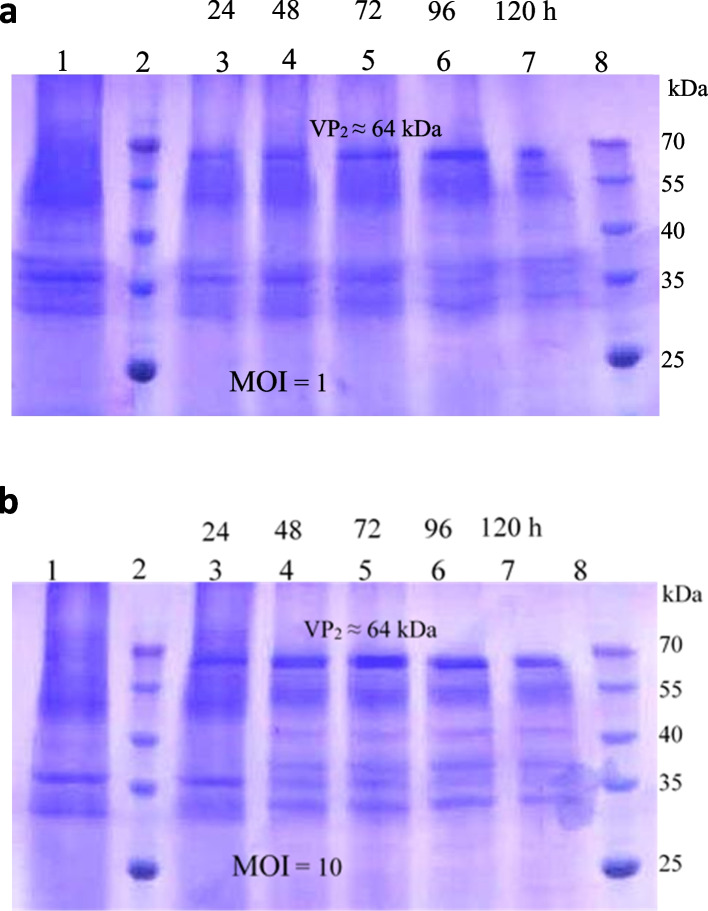
SDS-PAGE results of the proteins extracted from the *Sf9* cells infected by the recombinant baculoviruses encoding VP2. **a** The proteome of the cells infected with MOI of 1 (pfu/cell) and **b** the proteome of the cells infected with MOI of 10 (pfu/cell). Lane 1: The cell control, lanes 2 and 8: Molecular weight marker (Fermentas) and lanes 3–7: The protein samples taken at the harvest times of 24, 48, 72, 96, and 120 hpi, and the expected band (⁓64 kDa) corresponding to the recombinant expressed VP2

In the cells infected with MOI of 10 pfu/cell, at the harvest times of 24, 48, and 72 hpi, the expression of recombinant VP2 showed a significant increase, while the total protein content in these cells decreased with a gradual increase in cell death and from the fourth day onwards, with increasing cell death, the expression of recombinant VP2 also decreased. Figure 3 shows the results of the qualitative evaluation of the expressed recombinant VP2 as well as total protein content in flasks inoculated with MOIs of 1 and 10 (pfu/cell) in the mentioned schedule (compared to the control sample) using the SDS-PAGE method. The control sample used is related to the first 24 h, and the protein content of other samples has been compared.

**Fig. 4:**
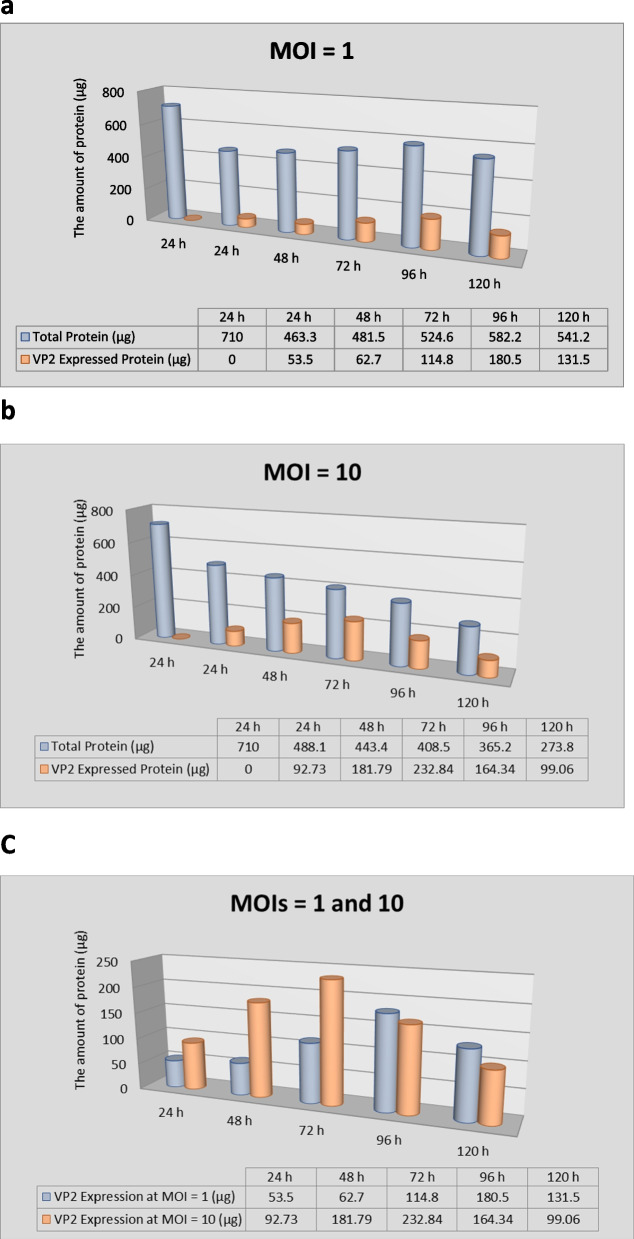
**a** Quantitative analysis of the expressed recombinant VP2 as well as the total protein content in the flasks infected with MOI of 1 (pfu/cell) at the harvest times of 24, 48, 72, 96, and 120 hpi. **b** Quantitative analysis of the expressed recombinant VP2 as well as the total protein content in the flasks infected with MOI of 10 (pfu/cell) at the mentioned times. **c** Comparison of quantitative analysis of the recombinant VP2 expression rate in the flasks infected with two MOIs of 1 and 10 (pfu/cell) at the mentioned time point. The results showed that the optimal expression of VP2 is related to the flask inoculated with MOI of 10 (pfu/cell) at the harvest time of 72 hpi

To calculate the expression percentage of recombinant VP2 protein, the gel documentation system (with BioDoc Analyze 2.2 software) was used (data not shown), and then, the amount of recombinant protein in each flask was calculated. The amount of recombinant VP2 protein obtained from the culture in the T25 flask inoculated with recombinant baculovirus with MOI of 1 (pfu/cell) at 24, 48, 72, 96, and 120 h was equal to 53.5 μg, 62.7 μg, 114.8 μg, 180.5 μg, and 131.5 μg, respectively, and with MOI of 10 (pfu/cell) at 24, 48, 72, 96, and 120 h was equal to 92.73 μg, 181.79 μg, 232.84 μg, 164.34 μg, and 99.06 μg. The maximum expression rate of the *VP2* gene with MOIs of 1 and 10 (pfu/cell) was related to the harvest times of 96 and 72 hpi and equivalent to 31% and 57%, respectively. Figure 4a and b show the results of quantitative evaluation of the recombinant VP2 expression rate as well as the total protein content in flasks inoculated with MOIs of 1 and 10 (pfu/cell) in the mentioned schedule (compared to the control sample).

Comparing the results of quantitative analysis of the recombinant VP2 expression rate in the flasks infected with two MOIs of 1 and 10 (pfu/cell) at the mentioned times showed that the highest amount of expression with appropriate quality of the recombinant protein is related to flask inoculated with MOI of 10 (pfu/cell) at the harvest time of 72 hpi. The result of this comparison is shown in Fig. 4c.

### Evaluation of the accuracy of the expressed recombinant VP2 by western blotting

**Fig. 5:**
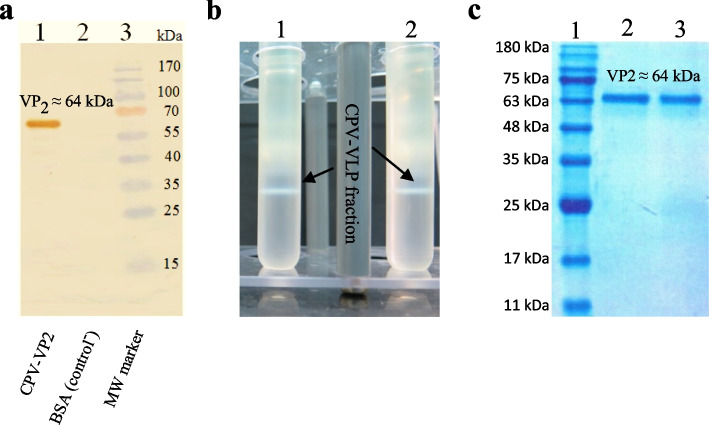
**a** The result of western blotting for recombinant expressed VP2, Lane 1: The VP2 band (~64 kDa) confirmed by using a specific mouse monoclonal antibody against this protein, Lane 2: The negative control (BSA), Lane 3: Prestained protein ladder (MyBioSource, USA). **b** The protein fraction resulting from the accumulation of intended VLP nanoparticles in tubes 1 and 2 containing discontinuous cesium chloride gradient after ultracentrifugation. **c** SDS-PAGE result of the purified VLPs, Lane 1: Molecular weight marker (Sinaclon, Iran), Lanes 2 and 3: The expected protein band (~64 kDa) related to VP2 subunits resulting from disassembly of the purified VLPs

With the proper expression of the recombinant VP2, in order to confirm the accuracy of the expressed recombinant protein, the western blotting was performed using a mouse monoclonal antibody against CPV-VP2 and the result of this evaluation showed the accuracy of the recombinant protein expressed in insect cells (Fig. 5a).

### Production of recombinant VP2 under the optimal conditions, extraction, and purification of formed biological virus-like nanoparticles (CPV-VLPs)

After optimization of MOI and harvest time of inoculated *Sf9* insect cells, by selecting the optimal conditions (MOI of 10 pfu/cell and the harvest time of 72 hpi), the higher levels and sufficient quantities of the recombinant VP2 were produced for VLP formation in insect cells based on the self-assembly of VP2 protein monomers. The VLPs were extracted and purified by chemical and physical lysis methods, followed by two-stage ultracentrifugation using 20% sucrose and discontinuous concentration by a two-layer gradient of 32% and 59% cesium chloride and the protein fraction resulting from the accumulation of the desired nanoparticles (CPV-VLPs) in the tubes containing a discontinuous cesium chloride gradient was harvested after ultracentrifugation (Fig. 5b). After dialysis in order to change the buffer, the purified sample was concentrated using Amicon Ultra centrifugal filter with a membrane NMWL of 30 kDa (cutoff = 30 kDa). According to the results of the Bradford assay, the concentration of purified VLP in the final sample obtained from 30 flasks of T75 was equal to 0.85 μg/μl and the amount in the final volume of 1.5 ml was 1.27 mg, which is equivalent to approximately 6% of the total amount of the recombinant expressed VP2 (20.96 mg). Figure 5c shows the protein band (related to VP2 subunits) of the final product by SDS-PAGE after the steps of purification, dialysis, and concentration.

### Investigation of the structural integrity of the purified VLPs by TEM

**Fig. 6:**
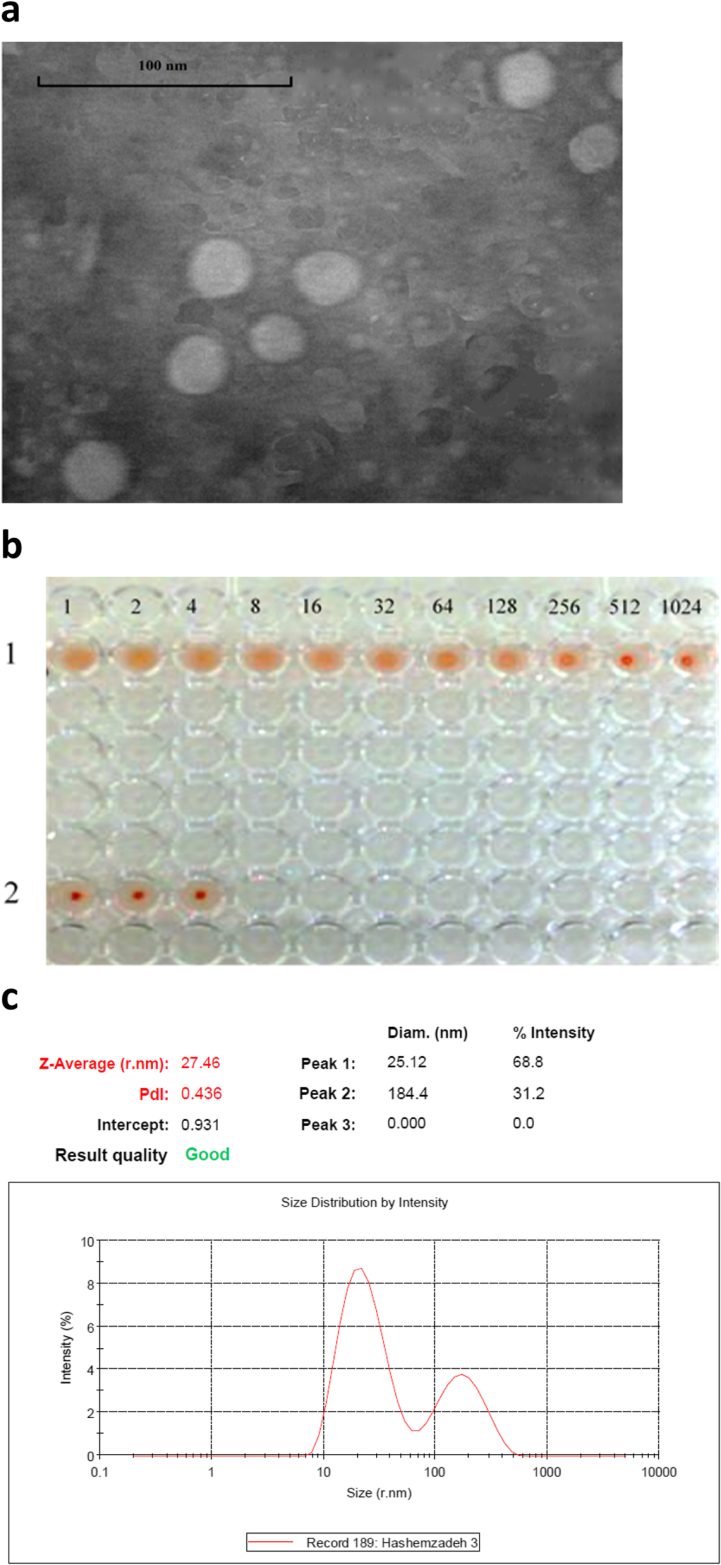
Evaluation results of the quality and structural integrity of the purified VLPs by TEM, HA, and DLS. **a** The purified VLPs imaged by TEM with a magnification of 120,000 X. **b** HA test in a 96-well *U* shaped plate. Row 1: The results indicate the strong hemagglutination in the dilutions of 1, 2, 4, 8, and 16 and weakening of hemagglutination in the dilutions of 32, 64, and 128, and finally, the negative hemagglutination in the dilutions of 256, 512, and 1024. In total, the hemagglutination property of the produced VLPs indicates the quality and structural integrity of these nanoparticles. Row 2: The negative control of HA containing PBS and RBC without the presence of the VLP. **c** Calculation of the size distribution of CPV-VLP nanoparticles by DLS technique. This analysis shows that 68.8% of the formed nanoparticles had a size of about 25.12 nm and 31.2% of these nanoparticles had a size of about 184.4 nm

The morphology and structural integrity of the purified VLPs were evaluated using transmission electron microscopy (TEM) and negative staining. The result of this microscopic analysis is shown in Fig. 6a which confirmed the structural integrity of the VLPs produced.

### Evaluation of the quality and structural integrity of the purified VLPs by HA

As described previously, since the VLP has a structure similar to a natural capsid when formed, it is expected to have similar hemagglutination properties. This is why the VP2 monomers alone do not agglutinate the related RBCs. Therefore, in this study, HA was used to evaluate the quality and structural integrity of the formed VLPs and the results showed the preservation of the integrity and also the quality of the produced nanoparticles (Figure 6b).

### Investigation of the size distribution of the purified nanoparticles by the DLS technique

The result of VLPs size analysis with Malvern Zeta-sizer (Fig. 6c) showed that 68.8% of the formed nanoparticles had a size of about 25.12 nm and 31.2% of these nanoparticles had a size of about 184.4 nm which is related to the VLP nanoparticles accumulated in the sample. The PdI ˂ 0.5 (0.436) indicated the homogeneity and uniformity of the formed nanoparticles.

The numerical values obtained from this evaluation have been calculated by the relevant software with the formula, based on the amount of loaded protein and its concentration.

## Discussion

Among all examined nanoparticles, the VLPs are quite distinct. Important properties of these nanoparticles include intrinsically nanometric structure and the possibility of penetration into tissues such as cancer tissues; and having a uniform, regular, and symmetrical geometric shape; as well the same size; furthermore, any of the intrinsic controls on these nanoparticles cannot be found in inorganic particles and liposomes, as a result, particle size homogenization and purification require complex. Other properties of produced nanoparticles are high resistance of VLPs to environmental conditions and ease of transport and distribution of materials in the body, biocompatibility and non-toxicity, high production efficiency, high capacity of binding exogenous peptides to their functional groups, and high carrying capacity of drugs.

VLPs are one of the best tools for producing the next-generation vaccines (nano-vaccines), and the definitive future of many vaccines will be VLP-based. This is due to a number of factors, including the ease of VLP production compared to advanced reverse-genetic systems and vector-based vaccines as well as being a suitable alternative to dangerous infectious viral agents, dead and attenuated live vaccines that purifying toxic and virulent agents is crucial in their generation process, and furthermore, their structure is not necessarily the same as the natural structure; however, in the formation of the VLPs, it considers necessary to form a natural and expected structure, since the minimum subunits needed to form the natural structure are in use. Thereby, there is a lack of tools to form other similar and diverse structures. As a result, if the conditions for the formation of a suitable and ideal structure do not exist, there will any structure will be formed. One of the best choices in future viral vaccines is the VLP-based vaccines, since they have all the characteristics of a complete virus but lacks the genome and infectivity [1, 16]; moreover, the NOVAVAX is one of the most reputable vaccine manufacturing companies in the world and is currently conducting extensive research on the production of VLP for all types of infectious viruses such as COVID-19 (Nuvaxovid), HIV, RSV, and Ebola in the pre-clinical and clinical stages. Another application of these nanoparticles in vaccine production is their use as nano-substrates for the presentation of various epitopes to the immune system [17].

Due to the VLP feature, the produced nanoparticles can be used as nanocarriers in order to deliver drugs, nucleic acids for gene therapy, and desired molecules to target cells, specifically. These applications are important in the treatment of diseases such as cancer, especially in the case of the CPV-VLP, as of its inherent property of binding to cancer cells [18].

Most research in the world on VLP production has been done so far on vaccine development for seasonal influenza viruses and other various strains such as H5N1 and H1N1 [19–23]. The first successful attempt in the world to generate VLP has been made against human papillomavirus (HPV), which causes cervical cancer, genital warts, and other rare types of cancer [16, 24–26], and currently, there are two VLP-based HPV vaccines in the market: Gardasil (or Silgard), which was approved by the Food and Drug Administration (FDA) and produced by Merck as well as Cervarix, which received this approval in 2009 and was produced by GlaxoSmithKline [26, 27].

Despite many efforts so far, the CPV-VLP has not yet been introduced to the market, although research on its initial production has been done [23, 28–31].

In 2006, Singh and colleagues used a traditional baculovirus system by homologous recombination method in *T. ni* insect cells to produce these VLPs [32]. According to the report, the production efficiency in the mentioned cell is about 0.5 mg per liter of suspended culture, which is extremely low. In our study, while using the convenient and low-cost expression system, the amount of the VLP produced was more than 5 mg per liter of monolayer culture, which can increase significantly by optimizing the purification process and using suspended culture. In our experiments, the *Sf9* cell was used instead of the *T. ni* cell which has a very high efficiency in the expression of recombinant proteins and the new Bac-to-Bac expression vector system with site-specific transposition mechanism (instead of traditional homologous recombination) was used to produce the recombinant baculovirus expressing VP2, which has much higher efficiency of recombination. However, the previous mentioned system requires time and cost to isolate recombinant and non-recombinant viruses and that the process is with low efficiency of plaque production method [32–36], while in the present study, these issues were solved and furthermore the quality of generated VLP was evaluated and confirmed. In Singh’s study, a single-stage ultracentrifugation method was used to separate and purify the VLP, which is due to the presence of impurities, the separation of the desired fraction from others is more crucial. According to the data of international standard tables and the buoyancy density of the desired particle [37], the use of a two-step method using sucrose and the discontinuous gradient of calcium chloride was found to be more suitable for the separation of these particles [38] and the experience gained in the present study showed the robust results.

In 2014, Xu et al. evaluated the self-assembly of virus-like particles of CPV from the capsid subunits expressed in *E. coli* for use as a vaccine [39]. In that study, most of the expressed VP2 proteins were produced in the form of inclusion bodies, which used the SUMO fusion motif as a solubilizing motif to increase the expression efficiency in the soluble form. After expressing SUMO-VP2 chimeric protein and its extraction and purification, they had to cut the motif added to the VP2 protein and re-purify the VP2, a process that reduced the efficiency of the system to generate CPV-VLP and required time and cost. In addition, the use of prokaryotic expression systems for expressing eukaryotic and viral proteins has important disadvantages.

One of the advantages and strengths of this study (in order to solve these problems) is the selection of a baculoviral expression vector system (BEVS) along with the appropriate expression host, as one of the most powerful and efficient eukaryotic expression systems [40], so that the resulting protein in these systems has a proper folding with a structure and function similar to natural protein [33], which is very important in the formation of VLPs [41]. Our previous studies also showed methods for producing recombinant fused proteins [42, 43]..

## Conclusion

Our study demonstrated that the BEVS using the *Sf9* insect cells on the basis of a new Bac-to-Bac expression system with a site-specific transposition mechanism is a suitable and efficient system for the production of CPV-VLP and the used method based on two-stage ultracentrifugation also was appropriate for purifying these nanoparticles. The VLPs produced can be used as the biologic nanocarriers to deliver drugs, nucleic acids, and desired molecules into the target cells such as human cancer cells, specifically. All assessments were conducted in accordance with ethical principles and under the supervision of the University's Ethics Committee (Ethic NO.IR.BMSU.REC.1400.037).

## Data Availability

Not applicable

## Abbreviations

CPV: Canine parvovirus
VLP: Virus-like particle
