# Correction: Biosynthesis of a VLP-type nanocarrier specifc to cancer cells using the BEVS expression system for targeted drug delivery

**DOI:** 10.1186/s43141-023-00485-x

**Published:** 2023-04-03

**Authors:** Mohammad Sadegh Hashemzadeh, Nariman Gharari

**Affiliations:** 1 Nanobiotechnology Research Center, Baqiyatallah University of Medical Sciences, Tehran, Iran; 2 Department of Molecular Biotechnology and Health Sciences, University of Turin, Turin, Italy

**Correction: J Genet Eng Biotechnol 21, 20 (2023)**

**https://doi.org/10.1186/s43141-023-00479-9**

Following publication of the original article [1], the author group has identified an error in Figs. 3, 4, and 6. The correct figures are given below.

**Fig. 3:**
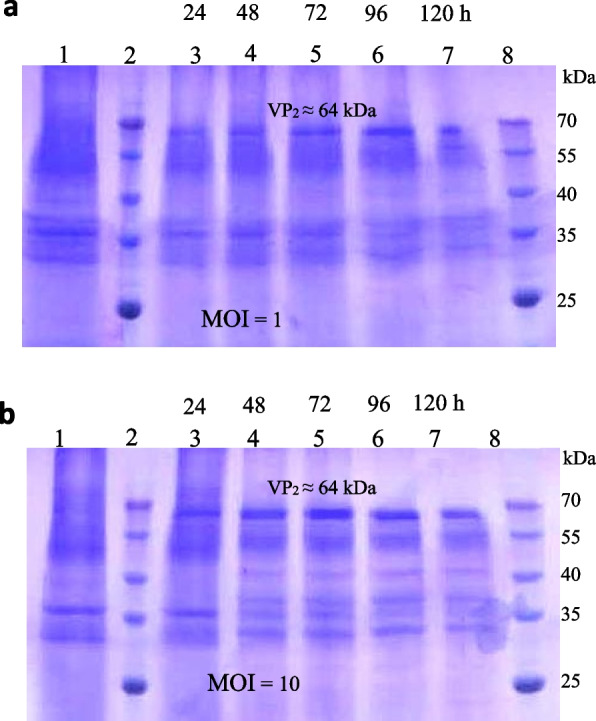
SDS-PAGE results of the proteins extracted from the Sf9 cells infected by the recombinant baculoviruses encoding VP2. **a** The proteome of the cells infected with MOI of 1 (pfu/cell) and **b** the proteome of the cells infected with MOI of 10 (pfu/cell). Lane 1: The cell control, lanes 2 and 8: Molecular weight marker (Fermentas) and lanes 3–7: The protein samples taken at the harvest times of 24, 48, 72, 96, and 120 hpi, and the expected band (⁓64 kDa) corresponding to the recombinant expressed VP2

**Fig. 4:**
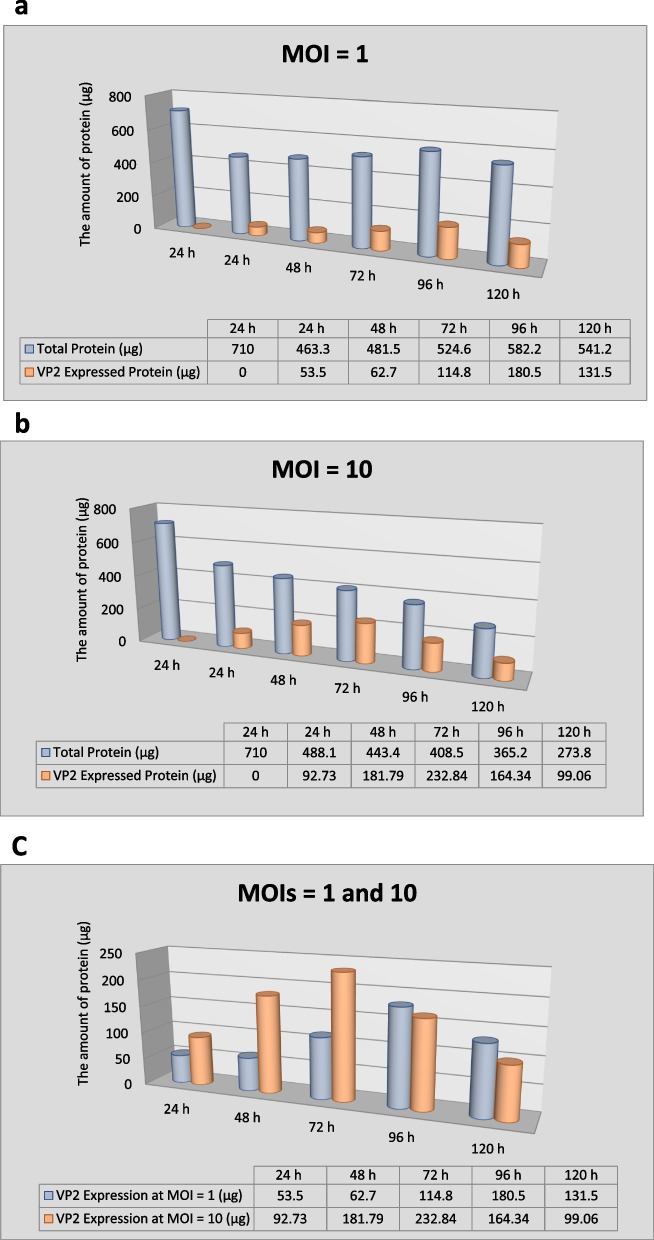
**a** Quantitative analysis of the expressed recombinant VP2 as well as the total protein content in the flasks infected with MOI of 1 (pfu/cell) at the harvest times of 24, 48, 72, 96, and 120 hpi. **b** Quantitative analysis of the expressed recombinant VP2 as well as the total protein content in the flasks infected with MOI of 10 (pfu/cell) at the mentioned times. **c** Comparison of quantitative analysis of the recombinant VP2 expression rate in the flasks infected with two MOIs of 1 and 10 (pfu/cell) at the mentioned time point. The results showed that the optimal expression of VP2 is related to the flask inoculated with MOI of 10 (pfu/cell) at the harvest time of 72 hpi

**Fig. 6:**
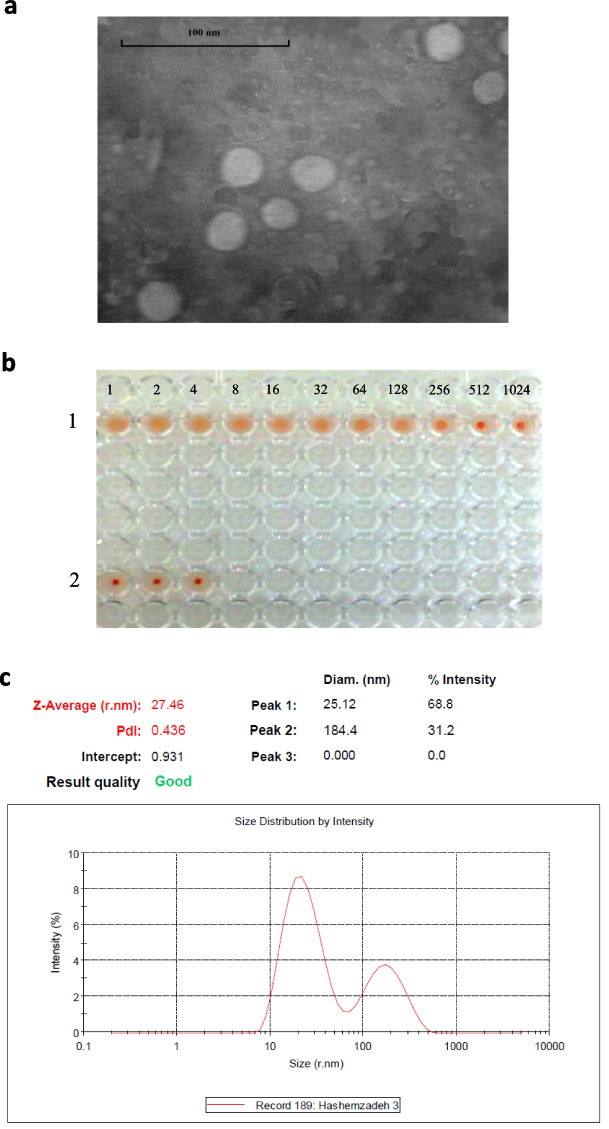
Evaluation results of the quality and structural integrity of the purifed VLPs by TEM, HA, and DLS. **a** The purifed VLPs imaged by TEM with a magnifcation of 120,000 X. **b** HA test in a 96-well U shaped plate. Row 1: The results indicate the strong hemagglutination in the dilutions of 1, 2, 4, 8, and 16 and weakening of hemagglutination in the dilutions of 32, 64, and 128, and fnally, the negative hemagglutination in the dilutions of 256, 512, and 1024. In total, the hemagglutination property of the produced VLPs indicates the quality and structural integrity of these nanoparticles. Row 2: The negative control of HA containing PBS and RBC without the presence of the VLP. **c** Calculation of the size distribution of CPV-VLP nanoparticles by DLS technique. This analysis shows that 68.8% of the formed nanoparticles had a size of about 25.12 nm and 31.2% of these nanoparticles had a size of about 184.4 nm

The original article [1] has been corrected.
